# Light microscopy applications in systems biology: opportunities and challenges

**DOI:** 10.1186/1478-811X-11-24

**Published:** 2013-04-11

**Authors:** Paul Michel Aloyse Antony, Christophe Trefois, Aleksandar Stojanovic, Aidos Sagatovich Baumuratov, Karol Kozak

**Affiliations:** 1 Luxembourg Centre for Systems Biomedicine (LCSB), University of Luxembourg, Esch-sur-Alzette, Luxembourg; 2 Interdisciplinary Centre for Security, Reliability and Trust (SnT), University of Luxembourg, Luxembourg City, Luxembourg; 3 Light Microscopy Centre (LMSC), Institute for Biochemistry, ETH Zurich, Zurich, Switzerland; 4 Medical Faculty, Technical University Dresden, Dresden, Germany

**Keywords:** Microscopy, Systems biology, Image analysis, Segmentation, Features, Machine learning

## Abstract

Biological systems present multiple scales of complexity, ranging from molecules to entire populations. Light microscopy is one of the least invasive techniques used to access information from various biological scales in living cells. The combination of molecular biology and imaging provides a bottom-up tool for direct insight into how molecular processes work on a cellular scale. However, imaging can also be used as a top-down approach to study the behavior of a system without detailed prior knowledge about its underlying molecular mechanisms. In this review, we highlight the recent developments on microscopy-based systems analyses and discuss the complementary opportunities and different challenges with high-content screening and high-throughput imaging. Furthermore, we provide a comprehensive overview of the available platforms that can be used for image analysis, which enable community-driven efforts in the development of image-based systems biology.

## Introduction

Humans are essentially a visual species. Most of our sensory neocortex is engaged in the processing of visual inputs that we gather from our surroundings. Not surprisingly, visualization techniques are at the heart of science and engineering [1]. One of the ultimate goals of systems biology is to elucidate relationships between molecular system states and higher order phenotypic traits. However, light scattering and other optical properties of living matter complicate the acquisition of informative images. For many decades, chemical fixation and the slicing of biological matter have been used to improve the stability and optical properties of samples. However, understanding living dynamic biological systems by examining fixed specimens is, at the best, a heuristic process.

The main challenge of the post-genomic era is understanding the rules governing dynamic biological systems. Current genomic tools in combination with advances in microscopy and computation facilitate *in vivo* observations of any genetic entity of interest. Recent progress in biotechnology, technology, and interdisciplinary cooperation provides more realistic insights into biological processes than ever before. With regard to systems biology, microscopy is a tool that connects multiple scales of biological complexity, ranging from molecules to populations. Recent progress in light microscopy allows for unprecedented insights into nanostructures, as well as unprecedented experimental throughput. In addition, high-resolution three-dimensional (3D) imaging of small, whole organisms is now feasible across time [2]. In turn, the progress in imaging technologies requires computer vision techniques for automated image analysis.

### Light microscopy opportunities in systems biology

**Figure 1:**
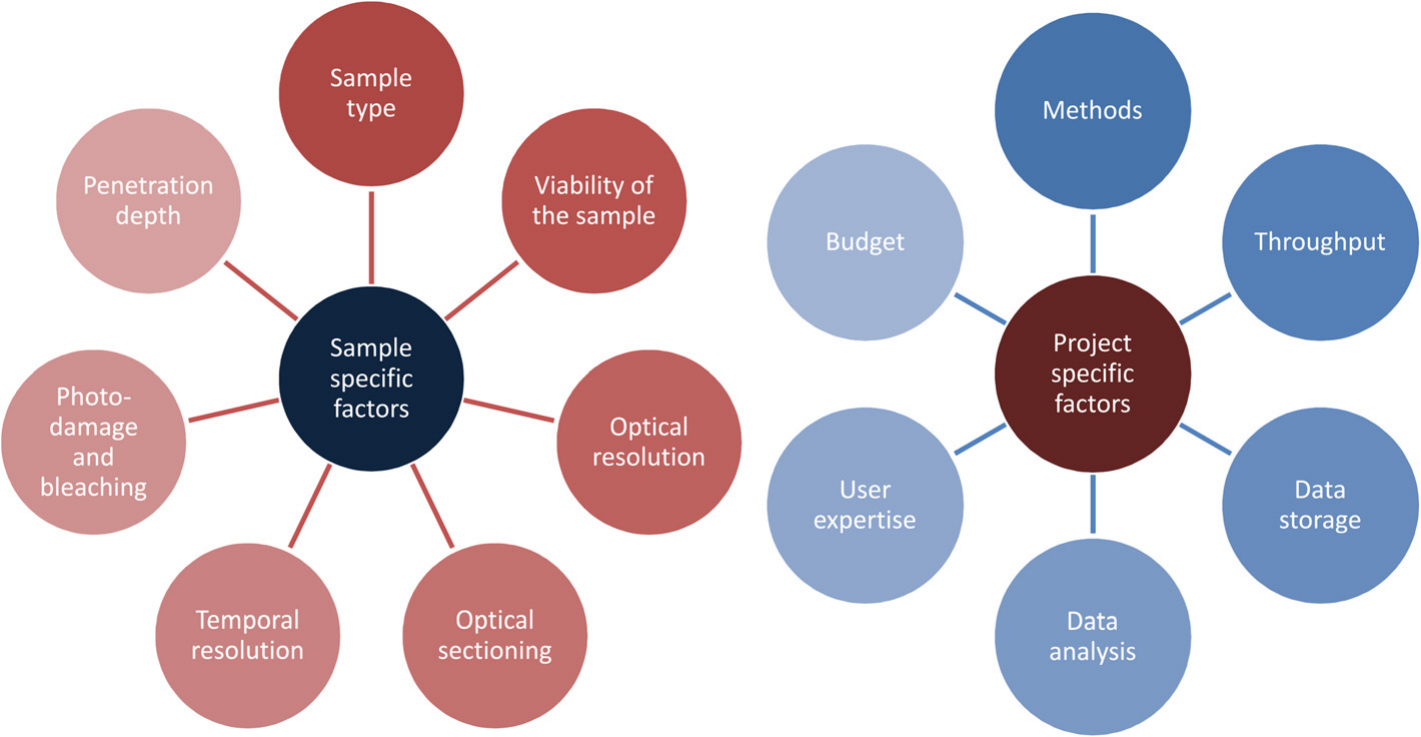
**Factors to be considered for the success of microscopy-based projects: **The development of highly specialized microscopes has improved the quality of raw data in image-based projects. However, optimal results are based on the choice of adequate imaging systems. A complete overview of available imaging technologies is beyond the scope of this review. However, as a guideline, the choice of an adequate microscope is based on sample- and project-specific factors. The optics of the microscope need to acquire images with adequate resolution and penetration depth, and a level of acceptable phototoxic stress needs to be considered for the illumination of the sample. At the level of project management, the needed throughput, which tends to be high in systems biology, needs to be considered, and an adequate image analysis infrastructure needs to be in place to avoid bottlenecks in image analysis and the interpretation of data.

Groundbreaking progress in technology during recent decades has leveraged the development of high-resolution microscopy [3-9]. In addition, improved understanding of chemical and physical properties of genetically encoded fluorescence markers has led to the optimization of live cell imaging applications and limited undesired experimental side effects [10]. Furthermore, the growing palette of available fluorescent proteins [11,12] and other fluorescent labels [13-16] has facilitated the imaging of a broad range of sample types, ranging from single molecules to whole organisms. On the other hand, most microscopes are highly specialized devices. Therefore, the selection of appropriate microscopes and data analysis tools requires the consideration of biological questions and sample properties (Figure 1). In the following sections we introduce biological systems ranging from single protein complexes to cell culture models and organisms of increasing complexity and give illustrative examples of appropriate light microscopy applications. In many cases, however, the shown techniques can be used for a whole range of sample types.

#### Molecular imaging

Molecular imaging is a discipline at the intersection between molecular biology and *in vivo* imaging. Optical molecular imaging can be used as a powerful tool for studying the temporal and spatial dynamics of biomolecules and their interactions [17], *in vitro* as well as *in vivo*.

On a purely molecular scale, imaging has for example provided an understanding of the rotational movement of F1-ATPase within ATP synthase [18]. The analysis of such highly structured macromolecular complexes of sizes and dynamics within nanometer and microsecond ranges, respectively, requires preliminary knowledge about molecular players. To observe the rotation under a microscope, Yasuda et al. [18] fixed subcomplexes of F1 on surface-bound beads and attached a fluorescently labeled actin filament to each γ subunit of ATP synthase. These structures were mounted on cover glasses. The *in vitro* addition of ATP finally triggered the continuous rotation of a few percentage of fluorescent actin filaments. At the time, these high-speed images obtained at single-molecule resolution were recorded on an 8-mm videotape. Since this work was published, new technologies have been developed to obtain even higher temporal and spatial data resolution [19]. However, sample preparations for such studies remain to be a manual and time-intensive endeavor [20].

Single molecule imaging in living matter provides the ability to study the molecular organization in cells and tissues by localizing specific molecules, such as RNA and proteins, in a native cellular context. However, many subcellular structures have dimensions lying below the diffraction limit of the visible light. Therefore superresolution microscopy techniques, allowing to look beyond the diffraction limit, such as PALM and STORM, are increasingly used for analyzing the organizational principles of molecular complexes and single molecules within living cells [21]. A central paradigm in systems biology is the aim for understanding biological networks including many different molecular factors. In classical fluorescence microscopy, however, the number of channels, which can be measured simultaneously, is limited by the spectral overlap between fluorophores. In this context it is important to note that recent developments have succeeded in increasing the number of molecular species that can be measured simultaneously. For example, Lubeck et al. [22] reported a method that drastically increases the number of simultaneously measurable molecular species by combining super-resolution microscopy and combinatorial labeling using mRNA barcodes with adjacent emitter/activator pairs. As a proof of concept, the authors analyzed the mRNA levels of 32 genes within a single yeast cell. Further improvements of this barcoding technology could potentially be used to perform -omics experiments at single-cell resolution, which could be a major milestone for systems biology.

From a holistic perspective, the mechanistic understanding of single molecular machines does, however, not allow for a complete understanding of higher level systems. Instead, it is important to study multiple scales of biological systems and identify potential signal transduction chains between molecules, cells, organs, and complex traits such as clinical syndromes. A major aim of modern systems biology and systems biomedicine is translational research, which develops clinical applications for improving patients’ quality of life [23]. However, before finding a clinical application, findings of *in vitro* experiments need to be validated in a more physiological context, such as molecular imaging in cell culture, live tissue culture [24], or a living brain [25-27].

#### Cellular Imaging

**Figure 2:**
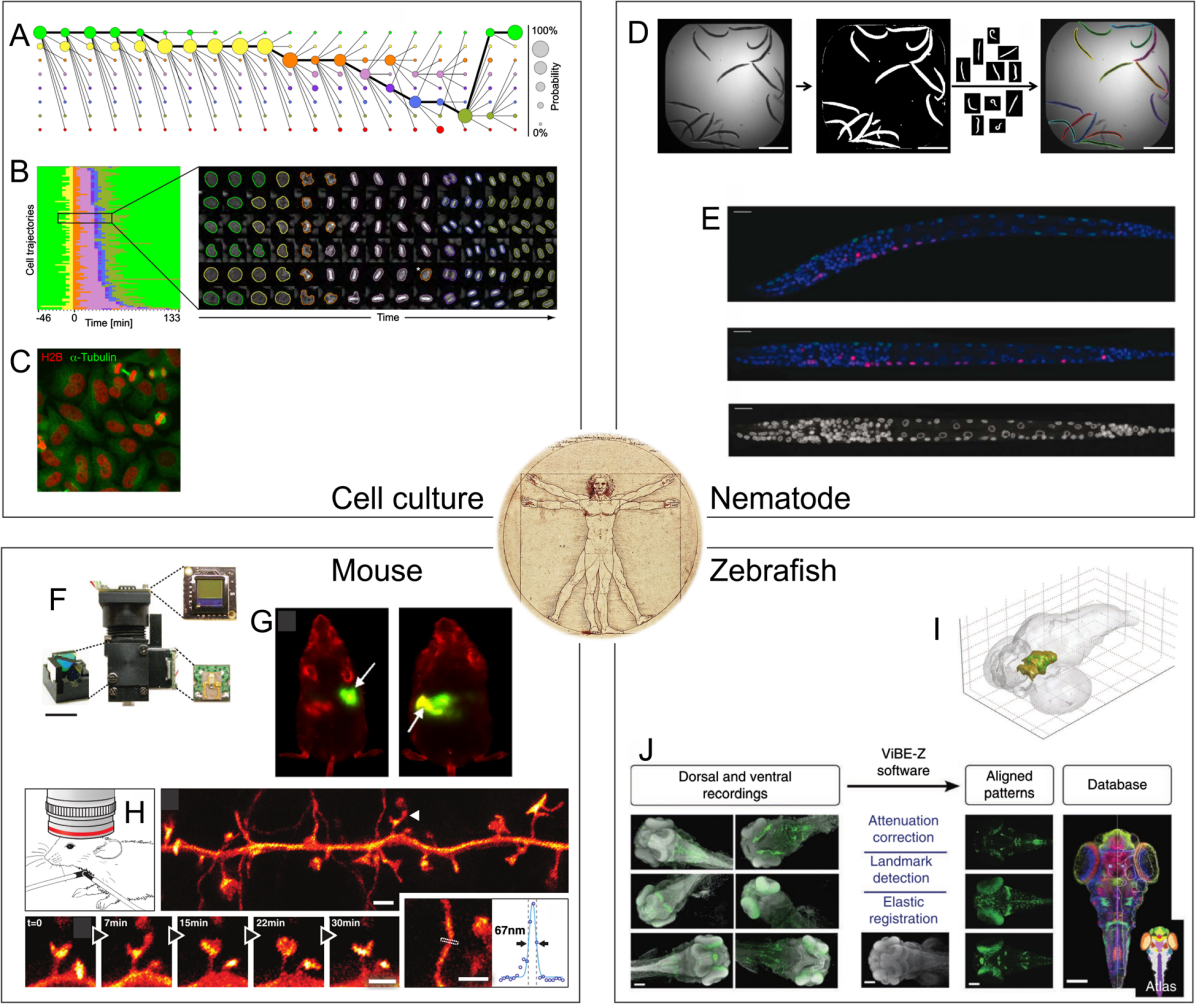
**Selected microscopy applications in systems biomedicine: **(**A**), (**B**), and (**C**) Analysis of mitotic events by hidden Markov modeling to evaluate mitotic phase transitions [33]. (**A**) Trellis diagram showing class prediction estimates for a given cell. (**B**) Event order map and example of time-series images. (**C**) Double-stained HeLa cells in different cell division states. (**D**) and (**E**) Nematode morphology analysis. (**D**) Automated segmentation of single worms [34]. (**E**) Straightening of single nematode datasets [35]. (**I**) and (**J**) Body atlas for zebrafish [36]. (**I**) TH-expressing zones are highlighted in green. (**J**) Registration of single image information into a zebrafish body-atlas database. (**F**), (**G**), and (**H**) *In vivo *imaging of mice. (**F**) Miniaturized microscope weighing 1.9 g [37]. (**G**) Dynamic analysis of the intestinal mucosal barrier function [38]. (**H**) Nanoscopy of dendritic spine dynamics in the brain of a living mouse [25]. All images were used with permission of the publishers.

The *in vitro* imaging of biophysical processes at the molecular scale requires time-intensive sample preparation, whereas the imaging of higher-scale processes (Figure 2) is often feasible at higher throughput, which is an important advantage in terms of statistical power and network analysis. Cell-based screening for biological or chemical compounds with biological effects is at the core of modern translational systems biology. High content screening (HCS) combines high-throughput microscopy with the automated extraction of a multitude of single-cell physiological features [28]. Automated microscopes equipped with an autofocus system [29,30] can be used to perform high-throughput experiments, in which the effects of hundreds of thousands of compounds or genetic perturbations are analyzed. The classical readouts of such image-based high-throughput screenings are fixed endpoints that can gather data from multiple image channels. While the lack of dynamical information is a constraint of endpoint measurements, both the possible high-throughput of endpoint measurements and the possibility to use antibodies that target intracellular antigens in fixed samples are valid arguments for choosing an endpoint analysis strategy [28]. In contrast to many biochemical assays, the resulting images of cell populations circumvent the limitations of population averages [31] by analyzing image data at the single-cell level [32]. However, the large volume of images produced by such high-throughput screening requires automated image analysis, including the identification and isolation of regions or objects of interest (segmentation) as well as the extraction of numerical intensity and morphology features.

In addition to single-cell information, light microscopy provides a path from rough static models to more refined dynamic models. Low- and medium-throughput automated microscopy can be used to acquire sequential image series of multiple samples and analyze the resulting kinetic data. The greatest strength of live cell imaging is its potential to assess the dynamics of cellular and even subcellular events. One example is a study by Chao et al. [39], in which the local translation of specific mRNAs was analyzed in single cells. With regard to cell populations, live cell imaging enables assessment of cellular heterogeneity and synchrony, which are important for understanding cellular differentiation [40-42], as well as local and global control mechanisms of transcription factors such as NF-kappaB [43,44].

Modern live cell imaging can build upon a whole arsenal of fluorescence-based methods that can be used to quantify the subcellular distribution of proteins, dynamics of subcellular shuttling processes, and molecular binding rates [4]. Using a highly tuned setup such as Förster resonance energy transfer (FRET) and fluorescence lifetime imaging microscopy, the dynamics of fast spatiotemporal protein-protein interactions can be observed at a molecular resolution [45]. However, the adequate interpretation of spatially resolved dynamic information requires more advanced analysis than steady-state images. In addition to segmentation, live cell imaging applications often require object tracking. Dynamic information can be highly relevant for translational research. For example, determining a correlation between hydrogel substrate elasticity and the migration of muscle stem cells could lead to the development of cell-based therapies for muscle-wasting diseases [46]. Classical tracking algorithms segment and track cells in a sequential approach by connecting neighboring time points. However, in contrast to classical tracking and cell lineage identification algorithms, improved algorithms that consider the entire image sequence, and prior knowledge (e.g., about mitosis and apoptosis) for annotating the best track and identifying the most likely cell lineage can be achieved [47].

The possibility of simultaneous measurement of a multitude of cellular properties or features gives HCS tremendous power and challenging complexity. Typical applications include screening for potential leads, molecules that are potential drug candidates, and genetic screening. Both approaches detect changes in cellular morphology resulting from molecular factors. While multiscale analysis in systems biology aims to connect molecular factors and phenotypic outcomes, HCS can be used for both top-down and bottom-up approaches [23]. Using HCS it is possible to analyze the effects of untested molecular factors on well-defined phenotypic outcomes or to classify multifactorial phenotypic traits for predicting underlying network states and pathways. Using HCS to predict potential pathways or the molecular targets of compounds identified in phenotypic screens is a hypothesis-generating approach that can provide new perspectives for understanding complex diseases with undiscovered pathogenic processes. In contrast, using HCS to validate potential drug targets is a more classical, hypothesis-driven approach, which requires more specific prior knowledge about phenotypic traits. One common example is RNA interference screening, which often focuses on a precisely defined phenotype [29].

The most advanced form of HCS combines bottom-up and top-down approaches. For example, HCS can combine genome-wide screening with a holistic view on a broad range of phenotypic features. A genome-wide RNA interference screen with highly advanced computational image processing performed by Neumann and Walter et al., used large-scale multifactorial phenotypic profiling from 2-day live cell imaging to successfully identify hundreds of human genes involved in diverse biological functions, including cell division, migration, and survival [48,49]. Importantly, this study also demonstrated the value of single-cell event-order analysis for investigations that aim to understand correlation and causality on a cellular scale.

The imaging of rare events such as cell division remains to be a challenging endeavor. One way to circumvent the manual selection of rare events is to use machine learning approaches to identify cellular events of interest. To fill this gap, Conrad et al. [50] developed Micropilot, a software that automates the identification of cell states and decision making for targeted image acquisition. Pre-filtering at the image acquisition level can lead to a loss of valuable information; hence, the applicability of pre-filtering depends on the type of pre-filtering and biological questions asked. For example, pre-filtering removes information from prior time points but allows for increased throughput of downstream event-order analysis at selected regions of interest.

#### Yeast

One primary goal of systems biology is to achieve a systems level of understanding of cellular genetics and physiology. The budding yeast, a simple and genetically tractable eukaryotic system, is a premier model organism for such functional genomic study [51]. Classical genomic screens in yeast have focused on specific morphological features such as cell size, cell shape, or bud site pattern [52,53]. In addition, the short lifespan of this model organism presents an interesting feature for research on aging. However, yeast cells in liquid culture are suspension cells. Budding results in an exponential increase in the number of daughter cells. The classical analysis of aging in short-lived budding yeast by taking snapshots of a single yeast cell throughout its entire lifespan [54,55] involves laborious manual dissections of daughter cells from larger mother cells. Recently, Lee et al. [56] described a microfluidics-based solution, in which cells were immobilized throughout their lifespan without accumulating daughter cells. As a result, the combination of microfluidics with microscopy drastically improved the workflow for image-based analysis of aging. Microscope-based cytometry is also a powerful tool with freely available software that quantifies fluorescence intensities in cellular resolution time series [57].

Similar to mammalian cell culture, yeast projects need to maximize the multiplicity of phenotypic readouts. The ready to use solution for multiparametric morphological analysis of yeast cells, CalMorph, is an image processing program that quantifies 501 cell morphology parameters in triple-stained yeast cells [58-60]. The automated phenotyping of subcellular events has successfully been used to identify drug targets based on morphological phenotypes of a reference mutant panel [61].

#### Caenorhabditis elegans

A pure cell culture-based analysis of gene regulatory networks is not sufficient for understanding signal transduction pathways, which can involve multiple regulatory mechanisms at different scales of biological complexity. Compared to yeast, the worm *Caenorhabditis elegans* has the advantage of being a multicellular animal model with higher genetic homology to humans. Furthermore, drug discovery screens with whole animals have the advantage of identifying compounds that modulate systemic phenotypes. Animal screens also have the potential to eliminate compounds with systemic toxicity earlier in the discovery process. The ability to conduct forward and reverse genetic screens in animal models such as *C. elegans* remains to be one of the most powerful experimental paradigms for understanding molecular pathways underlying human disease phenotypes [62]. The potential to analyze large numbers of isogenic animals through high-throughput and HCS for accessing different aspects of human disease phenotypes will certainly ensure an important role for this model organism in future oriented translational research [62-67]. With regard to imaging, its small size of only approximately 1 mm for adults and transparency at all stages of development are advantageous features. Finally, the possibility of flow sorting of worms by both size and fluorescence enables high-throughput experiments to be conducted [68].

One example of advanced image analysis in *C. elegans* is behavioral motility analysis. *C. elegans* can move through a wide range of environments, including crawling on substrates, swimming in fluids, and locomotion through microfluidic substrates. For classical locomotion analysis, each environment requires customized state-of-the-art image processing tools that rely on heuristic parameter tuning [69-80]. Sznitman et al. [81] recently proposed a so-called multi-environment model estimation framework that is versatile within various environments. In this image analysis process, statistical models for the background environment and nematode appearance are explicitly learned from a single image, which includes a nematode in its environment, and are used to accurately segment target nematodes.

Locomotive movements and complex morphological structures of the worms are of interest for multiscale approaches in systems biology, which aim to connect molecular events and organic states. Complete organisms such as *C. elegans* have more morphological features than simple cellular models. Green et al. [82] showed that steady-state phenotypic profiles of complex tissues such as gonads were sufficient for reconstructing a high-resolution genetic network of *C. elegans*. However, the relatively complex morphology presents a challenge for the comparative analysis of different worms. Image registration is a classical tool for resolving such problems. Recent developments in image processing can straighten *C. elegans* images, create an annotated 3D body atlas of the worm [35,83], and perform high-throughput morphological phenotyping of individual worms [34].

Advances in Bessel-beam technology and structured illumination microscopy promise even deeper insights, beyond the diffraction limit, into complex biological phenomena that require extended high-resolution time series in a multicellular context [7,84].

#### Zebrafish

In contrast to *C. elegans*, the zebrafish, also known as *Danio rerio*, belongs to the class of vertebrates, which is an advantage in the context of translational research. The zebrafish system with its small transparent larva can be used in diverse screening assays, including the analysis of development and organ function in living animals. In addition, genetic and chemical perturbation methods are well established [85,86]. Zebrafish can be used in small molecule screening, genetic screens, drug discovery, drug lead identification, and target identification [87,88]. However, the throughput of such screens decreases with the age and size of the fish. The main strength of this model lies in developmental biology applications [89] rather than applications related to aging.

In the context of imaging the central nervous system, high-resolution images of brain cells need to be acquired. Hence, blindly chosen fixed-field-views that lead to the potential omission of features of interest or low-resolution data of whole objects lacking cellular detail cannot fulfill this need. Stitching is an alternative of acquiring multiple fields of view at a high resolution for subsequent reassembling that can significantly increase imaging times and produce excessive and redundant data volumes. The problem of untargeted image acquisition patterns is a widespread issue that generally limits the efficiency of HCS assays. However, custom algorithms can solve this problem by automatically identifying predefined regions such as the fish brain, and triggering targeted high-resolution captures [90]. However, for interpreting brain phenotypes, the data from single fish need to be mapped to a standard brain map to facilitate the statistical evaluation of replicate zebrafish brains. This registration problem can be solved with the Virtual Brain Explorer (ViBE-Z), which is a software tool that maps cellular gene expression data to a 3D standard larval zebrafish by using a fluorescent stain of cell nuclei for image registration [36].

#### Mouse

Compared to previously described animal models, mouse models only enable moderate experimental throughput. Due to the optical properties of mice, immunohistochemistry remains a gold standard method in this field. One common strategy for increasing the experimental throughput is the use of tissue arrays [91]. Notably, modern image analysis tools can assist in the evaluation of the resulting colored tissue images [92,93].

Recently, evolved imaging techniques and image analysis tools have enabled non-invasive experimental workflows providing statistically relevant amounts of data. Near-infrared fluorescent optical imaging agents, which maximize the depth of tissue penetration, can be used for non-invasive whole mouse imaging, thus enabling the analysis of the presence and evolution of internal markers for disease progression [38,94]. Recent progress in image analysis has also been useful in the behavioral studies of mice; video tracking can be used to analyze the explorative behavior of mice [95]. For example, MiceProfiler is an open-source software that tracks and models the behavior of untagged mice [96].

The *in vivo* observation of live neurons is a useful approach because these cells perform their basic function of information processing by connecting with their neighbors. One way of monitoring the cellular dynamics of living neurons in mouse tissue is to use hippocampal slices of 5- to 7-day-old mice [97,98]. However, observing cellular dynamics in living mice is a more challenging endeavor. Berning et al. [25] used custom stimulated emission depletion microscopy to observe neurons and the movement of dendritic spines in the cerebral cortex of a living mouse [25]. This method was very invasive as optical access was provided by a glass-sealed hole in the skull of the anaesthetized and immobilized mouse. However, intravital microscopy is relevant for translational research, and significant technological progress has been made in recent years [99-101]. Two major limitations of classical intravital microscopy are the limited optical penetration depth and immobilization of mice; however, these limitations can be overcome by using miniaturized implantable microscopes [37,102-108].

### The challenge of quantitative image analysis

A central goal of image analysis is the conversion of microscopic images into biologically meaningful quantitative data. However, the amounts of image data produced using modern systems biology are very vast for manual analysis; hence, the development of automated image analysis tools is essential. Due to the complexity and size of modern imaging data, the computational analysis of biological imaging data has already become a vital emerging sub-discipline of bioinformatics and computer vision [109]. Research using multiparametric imaging data relies heavily on computational approaches for image acquisition, data management, visualization, and correct data interpretation [110-112]. The typical functions of dedicated computer vision systems are data pre-processing, image segmentation, feature extraction, and decision making [113,114]. Over the past 20 years, a myriad of commercial (Table 1) and open-source (Table 2) image analysis and data management tools have evolved [112,114]. In this review, we focus on open-source solutions, which facilitate community-driven efforts in the development of image analysis.

**Table 1:** Commercial software tools for image acquisition, processing, and analysis

**Product name**	**Supplier**	**3D rendering**	**Movie generation**	**Deconvolution**	**Multi-core**	**Editing**	**Tracking of objects**	**Segmentation**	**Large datasets**	**High-throughput**	**Mesh generation**	**MacOS X**	**Linux/Unix**	**Windows 32 bits**	**Windows 64 bits**	**Data management**	**Web-based access**	**Extendable**	**Main purpose**	**Link**
Able Image Analyser	Mu Labs	No	No	No	No	Yes	No	Yes	No	No	No	No	No	Yes	Yes	No	No	No	Analysis	http://able.mulabs.com/index.html
Acapella	PerkinElmer	No	Yes	No	Yes	Yes	No	Yes	Yes	Yes	No	No	Yes	Yes	Yes	No	No	Yes	Analysis	http://www.perkinelmer.com/pages/020/cellularimaging/products/acapella.xhtml
AcuityXpress	Molecular Devices	No	No	No	No	No	No	Yes	Yes	Yes	No	No	No	Yes	Yes	Yes	No	No	Storage/Analysis	http://www.moleculardevices.com/Products/Software/High-Content-Analysis/AcuityXpress.html
Amira	Vsg	Yes	Yes	Yes	Yes	Yes	Yes	Yes	Yes	No	Yes	Yes	Yes	Yes	Yes	No	No	Yes	Analysis	http://www.vsg3d.com/amira/overview
Aphelion Dev	ADCIS	No	Yes	No	No	Yes	Yes	Yes	No	No	No	No	No	Yes	Yes	No	No	Yes	Analysis	http://www.adcis.net/en/Products/Aphelion-Dev-4.x/Overview.html
AutoQuant	MediaCybernetics	Yes	Yes	Yes	Yes	Yes	Yes	No	No	No	No	No	No	Yes	Yes	No	No	No	Processing	http://www.mediacy.com/index.aspx?page=AutoQuant
AxioVision for Biology	Zeiss	Yes	Yes	Yes	No	Yes	Yes	Yes	No	No	No	No	No	No	No	Yes	No	No	Acquisition/Analysis	http://microscopy.zeiss.com/microscopy/en_de/products/microscope-software/axiovision-for-biology.html
Clemex Vision PE	CLEMEX	Yes	No	No	No	Yes	No	Yes	No	No	No	No	No	Yes	Yes	No	No	Yes	Acquisition	http://www.clemex.com/en/Products/Multipurpose-Image-Analysis/Clemex-Vision-PE/Description
Columbus	PerkinElmer	No	No	No	Yes	No	No	Yes	Yes	Yes	No	Yes	Yes	Yes	Yes	Yes	Yes	No	Storage/Analysis	http://www.perkinelmer.com/pages/020/cellularimaging/products/columbus.xhtml
Developer XD	Definiens	Yes	Yes	No	Yes	Yes	Yes	Yes	No	Yes	No	No	No	Yes	Yes	No	No	No	Analysis	http://developer.definiens.com/overview.html
Digimizer	MedCalc Software	No	No	No	No	Yes	No	Yes	No	No	No	No	No	Yes	Yes	No	No	No	Analysis	http://www.digimizer.com/
eCELLence	Glance	No	No	No	No	No	No	Yes	No	No	No	No	No	Yes	No	No	No	No	Cell Counting	http://www.gvt.it/ecellence
GSA Image Analyser	GSA	No	No	No	No	Yes	No	Yes	No	No	No	No	No	Yes	Yes	No	No	No	Analysis	http://image.analyser.gsa-online.de/
Huygens Software	SVI	Yes	Yes	Yes	Yes	Yes	Yes	Yes	Yes	Yes	No	Yes	Yes	Yes	Yes	No	No	Yes	Processing	http://www.svi.nl/HuygensSoftware
Image-Pro Premier	MediaCybernetics	Yes	Yes	No	No	Yes	Yes	Yes	No	No	No	No	No	Yes	Yes	No	No	No	Analysis	http://www.mediacy.com/index.aspx?page=IP_Premier
imageWarp	A&B Software	No	Yes	No	Yes	Yes	Yes	Yes	No	No	No	No	No	Yes	Yes	No	No	Yes	Analysis	http://www.imagewarp.com/index.html
Imago	MayaChitra	No	Yes	No	No	No	No	Yes	Yes	No	No	No	No	Yes	Yes	Yes	No	No	Analysis	http://mayachitra.com/imago/index.html
Imaris	Bitplane	Yes	Yes	No	Yes	Yes	Yes	Yes	Yes	No	No	Yes	No	Yes	Yes	No	No	Yes	Analysis	http://www.bitplane.com
IN Cell Investigator	GE Healthcare	No	Yes	No	No	Yes	Yes	Yes	No	No	No	No	No	Yes	Yes	No	No	Yes	Analysis	http://www.biacore.com/high-content-analysis/product-range/Overview/IN_Cell_Investigator/product_information/index.html
IN Cell Miner HCM	GE Healthcare	No	No	No	No	No	No	Yes	Yes	Yes	No	No	No	Yes	Yes	Yes	No	No	Storage	http://www.biacore.com/high-content-analysis/product-range/Overview/IN_Cell_Investigator/data_management/index.html
iSolution DT	i-Solution	Yes	Yes	No	No	Yes	Yes	Yes	No	No	No	No	No	Yes	Yes	No	No	No	Analysis	http://www.imt-digital.com/english/html/productsIMT.php
LAS Image Analysis	Leica	No	Yes	No	No	Yes	No	Yes	No	No	No	No	No	Yes	Yes	No	No	No	2D Analysis	http://www.leica-microsystems.com/products/microscope-imaging-software/life-sciences/las-easy-and-efficient/details/product/leica-las-image-analysis/
MetaMorph	Molecular Devices	Yes	Yes	Yes	Yes	Yes	Yes	Yes	No	No	No	No	No	Yes	Yes	No	No	Yes	Acquisition/Analysis	http://www.moleculardevices.com/products/software/meta-imaging-series/metamorph.html
Pax-it!	MIS	No	Yes	No	No	Yes	No	Yes	No	No	No	No	No	Yes	Yes	Yes	No	No	Storage/Analysis	http://www.paxit.com/paxit.asp
SlideBook	3i	Yes	Yes	No	No	Yes	Yes	Yes	No	No	No	No	No	Yes	Yes	No	No	No	Analysis	https://www.slidebook.com/
softWoRx Suite	Applied Precision	Yes	Yes	Yes	Yes	Yes	Yes	Yes	No	No	No	No	No	Yes	Yes	Yes	No	No	Visualization	http://www.api.com/softworx-suite.asp
Stream	Olympus	Yes	Yes	No	No	Yes	No	Yes	No	No	No	No	No	Yes	Yes	Yes	No	No	Storage/Analysis	http://www.olympus-ims.com/en/microscope/stream/
Volocity 3D	PerkinElmer	Yes	Yes	Yes	Yes	Yes	Yes	Yes	Yes	No	No	Yes	No	Yes	Yes	No	No	No	Analysis	http://www.perkinelmer.com/pages/020/cellularimaging/products/volocity.xhtml
ZEN 2011	Zeiss	Yes	No	Yes	No	No	No	No	No	No	No	No	No	Yes	Yes	No	No	Yes	Acquisition/Analysis	http://microscopy.zeiss.com/microscopy/en_de/products/microscope-software/zen-2011.html

**Table 2:** Open-source software tools for image processing and analysis

**Software**	**Class**	**Extendibility & Dimensionality**									**Description**	**References**	**Link**
		**Java**	**Matlab**	**C++**	**Perl**	**Python**	**R**	**2D**	**3D**	**nD**			
1C1V-Nauru	Analysis	Yes	No	No	Yes	Yes	Yes	Yes	Yes	Yes	Two-dimensional visualization of image-based screening data sets from high content screening	[115]	http://knime.org/
4D Viewer	Analysis	Yes	No	No	No	No	No	No	Yes	No	Plugin for ImageJ to visualize three-dimensional image stacks	[116]	http://3dviewer.neurofly.de/
ACME	Analysis	No	No	Yes	No	No	No	No	Yes	No	Membrane-based cell segmentation and morphology analysis that has been used for embryogenesis time-lapse datasets	[117]	https://github.com/krm15/ACME
Advanced Cell Classifier	Analysis	No	Yes	No	No	No	No	Yes	No	No	Data analyzer program using machine learning methods to evaluate cell-based high-content screens	[118]	http://acc.ethz.ch/
Bisque	Processing	No	No	No	No	Yes	No	Yes	Yes	Yes	Bisque (Bio-Image Semantic Query User Environment) was developed for the exchange and exploration of biological images and is widely used in plant biology	[119,120]	http://www.image.ucsb.edu/bisque
Bio-Formats	Processing	Yes	Yes	Yes	No	Yes	No	Yes	Yes	Yes	Standalone Java library for reading and writing life sciences image file formats	[121]	http://www.openmicroscopy.org/
BioImageXD	Analysis	No	No	Yes	No	Yes	No	Yes	Yes	No	Software for analyzing image-based high-throughput screening data	[122]	http://www.imagexd.net/
CellClassifier	Analysis	No	Yes	No	No	No	No	Yes	No	No	Matlab package of machine learning tools for the classification of cells or other biological objects	[123]	http://www.cellclassifier.ethz.ch
CellCognition	Analysis	No	No	Yes	No	Yes	No	Yes	No	No	Machine learning tool for time-resolved phenotype annotation that uses automatically extracted class transition probabilities to correct classification errors without user supervision	[33]	http://www.cellcognition.org/
CellExplorer	Analysis	No	Yes	No	No	No	No	No	Yes	No	Matlab code for a 3D digital atlas	[35]	http://penglab.janelia.org/proj/cellexplorer/
CellHTS Bioconductor	Analysis	No	No	No	No	No	Yes	Yes	Yes	Yes	Library for R-based analysis of cell based screens, visualization of screening data, statistical analysis, and connecting to other bioinformatics resources	[124]	http://www.bioconductor.org/
CellProfiler	Analysis	No	Yes	No	No	Yes	No	Yes	No	No	Image analysis platform designed for biologists without training in computer vision or programming for automated quantitative measurement of phenotypes from thousands of images	[125,126]	http://www.cellprofiler.org/
CellProfiler Analyst	Analysis	No	No	No	No	Yes	No	Yes	Yes	No	High-level data analysis platform that supports the CellProfiler framework. CellProfiler Analyst includes tools for classification, interactive data browsing, data mining, and visualization	[127,128]	http://www.cellprofiler.org/
EBImage	Analysis	No	No	No	No	No	Yes	Yes	Yes	No	Library of image analysis tools for the statistical programming environment R	[129]	http://www.bioconductor.org/packages/release/bioc/html/EBImage.html
FarSight	Analysis	No	No	No	No	Yes	No	Yes	Yes	Yes	Toolkit for Python-based multidimensional image analysis	[130]	http://farsight-toolkit.org
Fiji	Analysis	Yes	No	No	No	No	No	Yes	Yes	Yes	Software-engineering friendly ImageJ distribution with automated plugin management and the library ImgLib for type-, dimension-, and storage-independent representation of image data	[131]	http://fiji.sc/
iCluster	Analysis	No	No	No	No	No	No	Yes	Yes	No	Statistical tool that represents screening images in a spatial similarity layout	[132,133]	http://icluster.imb.uq.edu.au/
Icy	Analysis	Yes	No	No	No	No	No	Yes	Yes	No	Modern user and developer friendly open image informatics platform aiming to support extended reproducible research	[134,135]	http://icy.imageanalysis.org
Ilastik	Analysis	No	No	No	No	Yes	No	Yes	Yes	No	Pattern recognition-based image segmentation	[93,136]	http://www.ilastik.org/
ImageJ	Analysis	Yes	No	No	No	No	No	Yes	Yes	No	Java-based extendable package of microscope image analysis tools	[137-139]	http://rsbweb.nih.gov/ij/
ImageJ2	Analysis	Yes	No	No	No	No	No	Yes	Yes	Yes	Next generation of ImageJ	[112]	http://developer.imagej.net/
ImgLib2	Analysis	Yes	No	No	No	No	No	Yes	Yes	Yes	Java library for n-dimensional data representation and manipulation with a focus on image processing	[140]	http://imglib2.net
ITK	Analysis	No	No	Yes	No	No	No	Yes	Yes	Yes	The insight segmentation and registration toolkit (ITK) is a library, initially based on C++, that performs registration and segmentation	[141]	http://www.itk.org/
KNIME	Analysis	Yes	Yes	Yes	Yes	Yes	Yes	Yes	Yes	Yes	The Konstanz Information Miner (KNIME) is a workflow tool for the visual assembly and interactive execution of a data pipeline	[142]	http://www.knime.org/
mRMR	Analysis	No	Yes	Yes	No	No	No	Yes	Yes	Yes	Feature classifier for minimum redundancy maximum relevance feature selection	[143]	http://penglab.janelia.org/proj/mRMR/
OME	Processing	Yes	Yes	Yes	No	Yes	No	Yes	Yes	Yes	The Open Microscopy Environment (OME) provides file formats and metadata standards for microscope images	[144,145]	http://www.openmicroscopy.org/
OMERO	Processing	Yes	Yes	Yes	No	Yes	No	Yes	Yes	Yes	Visualization, multi user management, and analysis of biological microscopy images	[146,147]	http://www.openmicroscopy.org/
OMERO.searcher	Processing	No	No	No	No	No	No	Yes	No	No	Tool for content-based image retrieval	[148]	http://murphylab.web.cmu.edu/software/searcher/
OpenBis	Processing	Yes	No	No	No	No	No	Yes	Yes	Yes	Management system for biological information. The main goal is to support biological research data workflows from the source (i.e., the measurement of instruments and facilitating the process of answering biological questions using cross-domain queries against raw data, processed data, knowledge resources, and metadata)	[149]	http://www.cisd.ethz.ch/software/openBIS
OpenCV	Analysis	Yes	No	Yes	No	Yes	No	Yes	No	No	Library for feature extraction, tracking, and visualization in 2D plus time	[150]	http://opencv.org/
PatternUnmixer	Analysis	No	Yes	No	No	No	No	Yes	No	No	Machine learning tool used to determine the distribution of probes between different subcellular compartments	[151,152]	http://murphylab.web.cmu.edu/software/ PatternUnmixer2.0/
PhenoRipper	Analysis	No	Yes	No	No	No	No	Yes	No	No	Image block-based tool for the rapid exploration of high content microscopy images	[153]	http://www.phenoripper.org/
Vaa3D	Analysis	No	No	Yes	No	No	No	Yes	Yes	Yes	Extendible platform for 3D visualization-assisted image analysis	[154]	http://www.vaa3d.org/
VANO	Analysis	No	No	Yes	No	No	No	Yes	Yes	No	Object annotation system for 3D multicolor image stacks	[155]	http://vano.cellexplorer.org/
VisBio	Analysis	Yes	No	No	No	No	No	Yes	Yes	Yes	Visualization and analysis of multidimensional image data	[156]	http://loci.wisc.edu/software/visbio
VTK	Analysis	No	No	Yes	No	No	No	Yes	Yes	No	The visualization toolkit (VTK) is a library of C++ code for 3D computer graphics, image processing, and visualization	[157]	http://www.vtk.org/
Voxx	Analysis	No	No	Yes	No	No	No	Yes	Yes	No	Tool for fast, GPU-based 3D rendering	[158]	http://www.indiana.edu/~voxx/index.html
WND-CHARM	Analysis	No	No	Yes	No	Yes	No	Yes	Yes	Yes	Command line program for image-based feature extraction	[159]	http://code.google.com/p/wnd-charm/

Examples of microscopy developments requiring custom computational workflows for image acquisition include structured-illumination microscopy [160], super resolution microscopy [161-163], and Bessel-beam microscopy [5]. Some modern microscopes can produce up to 30 TiB of data per day [164]. However, the volume of images generated in systems biology is growing rapidly. As a result, the scalability of storage solutions and awareness for the need of image repositories and common file formats for imaging projects are increasing.

Research on image analysis has developed an entire ecosystem of image analysis tools. ImageJ [137-139], formerly known as NIH image, is a role model in the landscape of open-source tools for image analysis. Since its beginnings it has always been free and it became the most popular and widespread multipurpose image analysis tool. ImageJ has become successful because the scientific community can freely use it to focus on image analysis rather than on application programming. The concept of software extensibility by adding plugins is also useful for developers and end users. Furthermore, this concept has been adopted by more recently evolved platforms such as Fiji [131] and Icy [134,135]. The success story of ImageJ is continuing as the next-generation ImageJ2 software is currently under development (Table 2).

The 2 main challenges in image analysis in systems biology are the analysis of complex high-level structures such as whole organisms and the rise of experiments with ever increasing throughput. Imagery of large-scale biological systems such as embryos and brains requires state of the art algorithms for stitching, registration, and mapping to anatomical atlases. In addition to the extensible Vaa3D [154] and Fiji software packages, which are both established in this field, new tools such as TeraStitcher that can handle TiB-scale datasets have now emerged [165]. While the imaging of such high-level structures is typically conducted in a rather low throughput, partially automated workflows requiring a significant amount of user input are still quite common. In contrast, the amounts of images produced in high-throughput experiments are often increased by several orders of magnitude and cannot be manually analyzed. The challenge is to analyze data from HCS sets to a meaningful extent and in a reasonable amount of time. Several open-source packages for image analysis include functionality for machine learning-based cell classification. Some of these packages are CellProfiler [125,127], CellClassifier [123], and the R package EBImage [129], which provide workflows for fixed cell images.

CellProfiler can be used to address several application areas, including intensity and morphology measurements. In contrast to tools designed for fixed objects, CellProfiler can perform two-dimensional (2D) object tracking. Information about temporal coupling between cellular events is highly relevant for understanding the physiology of biological systems. Time-lapse imaging has emerged as a powerful tool for investigating dynamic cellular processes such as cell division or intracellular trafficking of labeled targets of interest. However, for the analysis of such high-throughput cinematography, only a few tools are currently available. CellCognition [33] is a freely available software platform that includes high-throughput batch processing and annotation of complex cellular dynamics such as the progression of single cells through distinct cell division states. In this platform, temporal hidden Markov modeling is used to reduce classification noise at state transitions and to distinguish different states with similar morphology. Briefly, CellCognition provides an analysis platform for live imaging-based screening with assays that directly score cellular dynamics [33]. BioImageXD [122], which is written in Python and C++, is leveraging the libraries VTK [157] and ITK [141]. As a result, BioImageXD, unlike CellProfiler and CellCognition, can offer options for 2D and 3D analyses by providing advanced batch-processing functions for multidimensional fluorescence image sets, including time series. In addition to built-in tools for visualization, colocalization analysis, segmentation, and tracking, the graphical user interface of BioImageXD facilitates the assembly of custom image analysis pipelines. The open-source design of the project, as well as the use of Python and gold standard file formats such as OME-TIFF, should further facilitate the evolution of this project for the community working on spatio-temporally resolved data [122].

An open-source software can foster productive collaborations between programming biologists and computer scientists interested in biology. However, an important challenge is to ensure the availability of analysis tools to the entire community of microscope users. The timely public availability of professionally programmed, easy-to-use, open-source tools for image analysis will depend on career opportunities for talented image analysis code writers [166], and the quality of these emerging tools will depend on good programming practices. Recently, Carpenter et al. [167] described usability criteria for image analysis software and advocated for usability as a more highly valued goal in broad-impact image analysis research. The authors emphasized that image analysis software should be user-friendly, modular, developer friendly, validated, and interoperable. Typically, the development of usable open-source software requires close collaborations between users and programmers, such that the resulting software does not suffer from the lack of software engineering expertise or real world applicability. An outstanding example of an open-source image informatics platform with very good usability is the most recently developed generalist image analysis platform Icy [134,135]. The main aim of this platform is to be developer friendly and facilitate timely and efficient collaborations as well as reproducible research. The software is built on Java but can also be used with the originally C++-based VTK and ITK libraries for native 3D visualization. The modern and well-organized user interface provides access to state-of-the-art image analysis tools and μManager-based [168] microscope control for live acquisitions with feedback. Furthermore, the writing of complete protocols is facilitated by a so-called workflow design tool, which represents individual processes as graphical blocks, and does not require any Java programming knowledge [135].

The creativity of researchers asking unprecedented scientific questions will continue to present challenges in image analysis that cannot be solved with a single software tool. Due to the common use of a variety of different software tools to acquire and analyze data, the connectivity and interoperability between these tools are crucial. Fortunately, many developers already understand this, and the most successful open-source image analysis platforms are explicitly developing ways to share data and code [112]. Finally, image analysis, with extraction of desired features, is needed but will not be sufficient for making biologically relevant conclusions. The extracted image-based features need to undergo further high-level data analysis. In turn, the analysis of extracted features and identification of relevant features can greatly improve with machine learning.

#### Machine learning

The increasing information content in image-based research poses new challenges for data interpretation. Multiparametric phenotype descriptors defined by a whole set of features, also known as phenoprints [29], can be used to cluster information contained in single pixels, single images, or whole screening datasets. However, machine learning-based classification can be used for image segmentation and high-level analysis of image-derived features [169].

Ilastik is an open-source tool based on user defined examples that train a machine-learning algorithm for identifying pixels of an image that belong to a class of interest [93,136]. This highly advanced segmentation approach is especially useful for images in which classical model-based segmentation gives poor results.

Machine learning can help classify image-based features obtained on image processing into biologically meaningful patterns. The following 3 general categories of tasks can be performed using image features: statistical comparisons, supervised learning, and unsupervised learning. In supervised learning, the user inputs prior knowledge by giving information, such as an annotation of an experimental condition, or indicating the concentration of a compound. In these cases, supervised machine learning can determine the most informative features for distinguishing the annotated biological patterns. Some examples are dose–response curves [170] and time points in time series [171].

CellCognition [33] was developed in the context of a genome wide screen for mitotic modulators. This tool utilizes a combination of explicitly coded image segmentation and supervised machine learning to automate the identification and annotation of mitotic stages. Considering the annotation of mitotic states in single cells, supervised learning means that the annotation of mitotic states must be performed manually for a small set of cells. This annotated training set is given to the learning algorithm to find a way of performing annotations on the remaining cells. For every cell in the training set and main dataset, the algorithm is given a set of input variables using which it labels the mitotic states. Formally, the learning stage consists of finding a mathematical function that maps input variables to the correct decision. Some readily available classifiers, including the support vector machine in its basic form, use linear decision functions. In CellCognition, however, support vector machines with a non-linear radial kernel are used. The main challenge in the setup of working classification algorithms is to define adequate features as input variables. Considering the type of attributes humans can use to perform the classification task may be helpful. Shape is an important attribute for classifying mitotic states (Figure 2B). However, shape cannot be readily quantified. Instead, CellCognition utilizes a set of quantitative features such as roundness for the classification process. The example of CellCognition illustrates that supervised machine learning can leverage the human interpretation of complex traits like shape and mathematical abstraction of such complex traits, which is needed for automated classification workflows in high-throughput projects.

In contrast to supervised machine learning, unsupervised learning such as cluster analysis can be used independently of prior knowledge to find groups within data. One example of unsupervised learning is the clustering of drugs by their effects [172]. Combinations of supervised and unsupervised learning are typically known as semi-supervised learning. A classical approach is to start with supervised learning to determine if the given features can be used to distinguish some major classes before using unsupervised learning to discover unknown subclasses of biological relevance [112].

#### Workflow systems

Workflow systems are recently beginning to emerge in image-based systems biology and give users more flexibility. These tools call applications such as image analysis tools and machine learning tools as components of an analysis pipeline. Workflow systems can be used to build virtual systems for image acquisition and can perform feature extraction and high-level data analysis without writing complex scripts. With the increasing need in sophisticated processing, image analysis, and high-level data interpretation, open-source workflow systems are gaining popularity. KNIME [142] is an open-source workflow system with a very broad set of domains that connects image analysis tools and other bioinformatics tools to create complex image processing and analysis workflows.

Some of the open-source image analysis tools can also be combined without using a workflow system. For example, CellProfiler, with its empowering integrative ability, can run an ImageJ macro or Ilastic machine-learning algorithm within the context of an automated image analysis pipeline. In the context of image-based systems biology, the main advantage of KNIME is that it can construct workflows beyond the direct interoperability of available image analysis tools. For example, KNIME can integrate the library ImgLib for n-dimensional image analysis from Fiji [131] into a workflow which was as yet missing this functionality.

#### Databases

The vast amounts of experiments, images, metadata, and extractable features in systems biology require relational databases. In HCS, there is an intrinsic need for user-friendly, scalable, and powerful information management systems. Data management platforms should enable users to collect, integrate, share, and publish data. In the scope of interoperability, these platforms should also be able to connect to data processing pipelines and workflow systems. The benefit of using open source databases is extendibility and the possibility of platform customization.

The Bio-Image Semantic Query User Environment (Bisque) [119] was developed for the exchange and exploration of biological images. The Bisque system supports several areas from image capture to image analysis and query. This platform is centered on a database of images and metadata. The integrated analysis tools allow high-level semantic queries to be made as well as comparisons of image content. Bisque was specifically designed to provide researchers with organizational and quantitative analysis tools for time-resolved multichannel 3D screening data. Images and metadata are organized with tags (i.e., name–value pairs) associated with an image. Typically, users locate images of interest by browsing through collections or searching with specific queries. The system has an integrated web image browser for the filtering, sorting, and ordering of images. The image organizer performs advanced sorting by hierarchical tag ordering. In addition, users can extend Bisque with data model and analysis extensions in order to adapt the system to local needs. The extensibility of Bisque stems from the following 2 core concepts: flexible metadata facility and an open web-based architecture.

The Open Microscopy Environment (OME) project [121,144,145] leverages imaging projects by focusing on the underlying need for common file formats. OME provides Bio-Formats, a tool that fully parses more than 120 proprietary image formats and converts proprietary metadata to the OME-XML data model. The OME-TIFF format is a container format for Tiff images with OME-XML metadata and the most widely used image format in community-driven projects. To ensure data integrity, Bio-Formats converts the proprietary file format metadata into a table of key-value pairs that is subsequently stored as an annotation on the imported image in the relational database OMERO [146]. OMERO was created to provide a single unified data management platform for image data generators and users. Briefly, OMERO uses a number of storage mechanisms for images and metadata and provides an application programming interface for using remoting image analysis tools that are based on C++, Python, Matlab, or Java. Recently added functionality also allows for organizing quantitative features in tables.

In HCS, it is crucial to keep track of quantitative features. OpenBIS [149] is a framework for constructing user-friendly, scalable, and powerful information systems for HCS data and metadata. OpenBIS allows users to collect, integrate, share, and publish image-based data and connect to data processing pipelines. This framework, which is built on a hierarchical structure ranging from project management layers to sample specific datasets, is easily extensible and specialized but not limited to imaging projects. OpenBIS is a flexible platform for handling images, structured metadata (e.g., sample annotations), and unstructured data (e.g., attached files), and is scalable to very large data.

A combination of databases with workflow systems such as KNIME can enable the integration of functionalities beyond the scope of classical image databases. For example, the KNIME node 1Click1View (1C1V) was developed to facilitate a link between large-scale image data sets from HCS and numeric data [115]. At the level of screening plates, 1C1V can be used to visualize quantitative features in form of heatmaps. Phaedra [173], another informatics tool connecting to KNIME, has been developed to support workflows for drug screening and target discovery. This tool can be used to plot dose–response curves, manage exclusion and annotation options, and perform cell classification, statistical quality controls, and reporting.

## Conclusions

Historically, microscopy has been a qualitative technique. However, due to advances in labeling and imaging methods, as well as computer vision and informatics, modern microscopy has widely improved the extraction of meaningful quantitative data from biological samples. Despite technological advances, a balance between experimental throughput, which is required for statistical significance, and the potential output of new biological knowledge needs to be found. Clear research plans and prior knowledge are key requisites for the progress of translational systems biomedicine. In many cases, image-based approaches can make major contributions. However, it is crucial to choose an adequate experimental model and use imaging technology that optimally fits with the chosen methods and properties of the sample. Given the vast variety of light microscopy applications in systems biology, the search for a universal image analysis tool that covers all the needs is often illusive. The correct approach is to focus on the production of high-quality raw data and use the flexibility of existing image analysis tools for integrating required image analysis and data processing workflows.

## Competing interests

The authors declare that they have no competing interests.

## Authors’ contributions

PA wrote the first version of the manuscript and incorporated all the contributions of the coauthors. CT prepared the tables of the manuscript, AS focused on the machine learning part, AB on microscopes, and KK helped to improve the review of workflow systems and databases. All authors read and approved the final manuscript.
